# Impact of low-dose aspirin on maternal haemoglobin levels during pregnancy: a protocol for a systematic review and meta-analysis

**DOI:** 10.1186/s13643-026-03219-5

**Published:** 2026-06-19

**Authors:** Nokwethemba Monica Ngcobo, Vino Dorsamy, Chauntelle Bagwandeen

**Affiliations:** 1 School of Laboratory Medicine and Medical Sciences, College of Health Sciences, University of KwaZulu-Natal, Durban, South Africa; 2 Department of Public Health Medicine, School of Nursing and Public Health, University of KwaZulu-Natal, Durban, South Africa

**Keywords:** Low-dose aspirin, Hemoglobin, Pregnancy, Preeclampsia, Obstetric haemorrhage, Systematic review, Meta-analysis

## Abstract

**Background:**

Aspirin, a cyclooxygenase (COX) inhibitor with anti-inflammatory and antiplatelet properties, is widely used during pregnancy to prevent preeclampsia (PE) and intrauterine growth restriction (IUGR). Despite its benefits, low-dose aspirin (LDA) has been linked to obstetric haemorrhage, including postpartum haemorrhage (PPH), and may influence haemoglobin (Hb) levels via its iron-chelating effects. The potential combined impact of LDA on Hb levels and pregnancy outcomes, however, remains unclear. This systematic review protocol aims to evaluate the effect of LDA on maternal Hb levels during pregnancy.

**Methods:**

Following PRISMA-P guidelines, comprehensive searches will be conducted in PubMed, Cochrane Library, Embase, Scopus, Web of Science, and ClinicalTrials.gov, as well as grey literature sources. Eligible studies will include randomised controlled trials (RCTs), cohort studies, and case-control studies reporting Hb levels in pregnant women using LDA. Two independent reviewers will perform screening, data extraction, and quality appraisal. Data will be synthesised using meta-analysis where feasible, with subgroup analyses to explore heterogeneity, and narrative synthesis for studies not suitable for pooling.

**Results:**

The systematic review will generate pooled evidence on how LDA affects maternal Hb levels and associated pregnancy outcomes such as PE and fetal growth.

**Conclusions:**

The findings may assist in refining clinical recommendations for LDA use in pregnancy by balancing its preventive benefits with potential risks related to anaemia, and haemorrhage.

**Systematic review registration:**

PROSPERO CRD42024556220.

**Supplementary Information:**

The online version contains supplementary material available at https://doi.org/10.1186/s13643-026-03219-5.

## Background

The prophylactic use of low-dose aspirin (LDA), also known as acetylsalicylic acid, spans an array of conditions including cardiovascular diseases, dementia, and certain cancers, making it one of the most universally prescribed medications globally [1]. Aspirin is a cyclooxygenase (COX) inhibitor with anti-inflammatory and antiplatelet properties. During pregnancy, LDA is commonly used to prevent preeclampsia (PE) and intrauterine growth restriction (IUGR) [2, 3]. Preeclampsia is a hypertensive disorder of pregnancy (HDP) characterised by increased blood pressure and proteinuria [4]. If left untreated, it can lead to organ damage, fetal growth restriction, and preterm birth [5, 6]. The therapeutic rationale for LDA lies in its capacity to alter the biochemical pathways implicated in the pathogenesis of PE. Aspirin modulates the COX enzyme pathway, leading to balanced production of thromboxane and prostacyclin, substances that play critical roles in placental blood flow regulation [7, 8]. Preeclampsia is associated with a more coagulable state marked by significant platelet activation [9, 10]. Low-dose aspirin inhibits the secretion of platelet-derived thromboxane A2 (TXA2), reducing vasoconstriction and platelet aggregation in the placenta, but does not change the secretion of prostacyclin 2 (PGI2), the most effective endogenous inhibitor of platelet aggregation [11]. This is a key step in preventing the early onset of PE. However, its impact on haemoglobin (Hb) levels and, by extension, anaemia during pregnancy remains underexplored. Maintaining optimal Hb levels during pregnancy is vital to ensure an adequate oxygen supply for both maternal and fetal well-being [12]. A decrease in Hb or red blood cells results in anaemia, a condition characterised by a reduced capacity of the blood to transport oxygen [13]. Globally, 37% of pregnant women and 30% of women 15–49 years of age are estimated to be affected by anaemia [14].

### *Rationale*

The use of LDA during pregnancy is associated with an increased risk of postpartum and intrapartum bleeding, postpartum haematoma, and intracranial haemorrhage [15]. This may result from its antithrombotic effect or excessive thromboxane inhibition as the effect of aspirin on COX-dependent prostaglandin is dose-dependent. Aspirin’s primary metabolite, salicylic acid, chelates iron, forming a complex that reduces iron bioavailability and can contribute to iron deficiency anaemia (IDA) [16–18]. Haemorrhage is the leading direct cause of maternal death worldwide and more than two-thirds of these cases are classified as postpartum haemorrhage (PPH) [19, 20]. Postpartum haemorrhage can cause a decrease in haemoglobin levels due to significant blood loss [21, 22]. A rapid decrease in Hb levels occurs because the body’s ability to replenish red blood cells is slower than the rate of blood loss during the PPH [23]. Globally, PPH accounts for 19.7% of maternal death, with 8% occurring in developed regions and nearly 20% occurring in developing regions [20]. Therefore, when advocating for the use of aspirin during pregnancy, the risk needs to be weighed against the potential benefits.

### Aims

The aim of this systematic review is to elucidate the impact of LDA on Hb levels in pregnancy and its subsequent effects on pregnancy outcomes such as PE, fetal growth, and birth weight. This will allow for evidence-based recommendations to refine anaemia management strategies, particularly in low- and middle-income countries (LMICs).

### Objectives

The specific objectives of this study are to

- Investigate the associations between the use of aspirin and maternal Hb levels.
- Identify the incidence of anaemia in pregnant women treated with aspirin.
- Determine whether changes in Hb levels influence specific pregnancy outcomes, such as the incidence of PE, fetal growth, and birth weight.

## Methodology

This protocol adheres to the recommendations outlined in the Preferred Reporting Items for Systematic Review and Meta-analysis for Protocols (PRISMA-P) guidelines. The results will be reported following the PRISMA 2020 statement, and the article screening and selection process will be depicted through a PRISMA 2020 flow diagram [24].

### Eligibility of the research question

The eligibility of the research question was determined using the Population Intervention Comparator Outcome (PICO) framework, as illustrated in Table 1.

**Table 1:** PICO framework for the eligibility of the research question

Population	Pregnant women
Intervention	Women prescribed LDA
Comparator	Placebo or no treatment
Outcomes	*Primary outcome* Change in Hb levels during pregnancy Incidence of anaemia Confounding factors *Secondary outcome* Incidence of PE Fetal growth rate, birth weight and preterm birth

### Inclusion criteria

Studies will be considered eligible if they meet the following PICO-based criteria:

- Population: pregnant women of any age and parity, with gestational age ≤ 28 weeks at the time of initiating LDA. No restrictions will be applied based on anaemia or hypertensive disorder risk.
- Intervention: use of LDA (75–162 mg daily) initiated between 12- and 28 weeks’ gestation during pregnancy. Studies must specify the dose, timing of initiation, and duration of aspirin therapy.
- Comparator: placebo, standard antenatal care, or no treatment.
- Outcomes: at least one of the following maternal outcomes must be reported:
  1. Change in Hb levels, assessed using venous blood samples analysed by standard laboratory haematology analysers.
    Baseline Hb: measurements obtained during early pregnancy (first or second trimester).
    Follow-up Hb: measurements taken in late pregnancy (third trimester) or at delivery.
  2. Incidence or severity of anaemia. Incidence of anaemia during pregnancy, defined as Hb levels below 11.0 g/dL in the first and third trimesters, and below 10.5 g/dL in the second trimester, in accordance with World Health Organization (WHO) criteria [25]. Where applicable, severity will be interpreted according to WHO thresholds:
    Mild anaemia: Hb (10–10.9 g/dL)
    Moderate anaemia: Hb (7–9.9 g/dL)
    Severe: Hb < 7 g/dL
  3. Confounders: potential confounders such as maternal nutrition, iron supplementation, chronic diseases and other medications will be noted to understand their influence on Hb levels.
  4. Occurrence of hypertensive disorders of pregnancy (HDP) defined according to International Society for the Study of Hypertension in Pregnancy (ISSHP) criteria, including:
    Gestational hypertension: new-onset hypertension after 20 weeks’ gestation.
    Preeclampsia: hypertension with proteinuria or other signs of organ involvement.
    Preeclampsia with severe features: such as severe headache, nausea, vomiting, visual disturbances, or seizures (eclampsia) [26].

#### Fetal outcomes

1. Fetal growth restriction is assessed using ultrasound parameters or birth weight percentiles relative to gestational age and sex, which are standard clinical proxies for impaired fetal growth.
2. Birth weight, defined as infant weight measured at delivery and reported in grams. Where applicable, birth weight will be analysed as a continuous variable and categorised according to standard thresholds (e.g. low birth weight < 2500 g).
3. Preterm birth, defined as delivery occurring before 37 completed weeks of gestation, assessed using gestational age at delivery.

#### Setting

No restriction will be placed on country, healthcare system, or level of care.

#### Study design

Eligible studies include randomised controlled trials (RCTs), cohort studies (prospective or retrospective), and case-control studies.

#### Language

There will be no restrictions on the language of publication. Articles not published in English will be translated for eligibility assessment using Google Translate or, where necessary, clarification will be sought from native speakers or professional translation services.

### Exclusion criteria

Studies will be excluded if they

- Are not available in full text, despite attempts to contact the authors
- Involve non-pregnant populations
- Are published as conference abstracts, case reports, reviews, or editorials
- Do not include an intervention group treated with low-dose aspirin and reporting on Hb outcomes.
- Lack a comparison or control group.

#### Information sources

A comprehensive search will be conducted using the following electronic databases: PubMed, CINAHL, EMBASE, EBSCO, Ovid Maternity and Infant Care, the Cochrane Database of Systematic Reviews, Google Scholar (for grey literature), Web of Science, SCOPUS, PsycINFO, and ClinicalTrials.gov.

In addition to electronic databases, we will also search the reference lists of included articles (backward citation chaining) and use forward citation tracking via Google Scholar to identify additional relevant studies.

#### Search strategy

Search terms will combine controlled vocabulary terms (e.g. Medical Subject Headings (MeSH) with free-text terms searched in titles, abstracts, and key words to maximise sensitivity and precision. The PubMed search strategy will be piloted using the following search string:

PubMed: ((“Aspirin”[MeSH Terms] OR "low-dose aspirin" OR "acetylsalicylic acid") AND (“Anaemia”[MeSH Terms] OR "haemoglobin" OR "hemoglobin" OR "blood iron levels" OR "iron deficiency"[MeSH Terms]) AND (“Pregnancy”[MeSH Terms] OR "pregnant women" OR "maternal" OR "pregnancy complications"[MeSH Terms] OR "hypertensive disorder of pregnancy"[MeSH Terms] OR "preeclampsia"[MeSH Terms] OR "pregnancy outcomes"[MeSH Terms] OR "obstetric hemorrhage"[MeSH Terms] OR "postpartum hemorrhage"[MeSH Terms])) as illustrated in Table 2 of Appendix 1.

This search strategy will be adapted for other electronic databases such as Cochrane Library, Embase, and Scopus. This approach improves both precision and recall of search results [27].

To ensure comprehensive coverage, grey literature sources will be searched, including ClinicalTrials.gov, OpenGrey, ProQuest Dissertations, and conference abstracts. These sources will help capture additional evidence, especially as published studies on the effect of LDA on Hb levels remain limited [28].

The search strategy will undergo peer review by an information specialist following the Peer Review of Electronic Search Strategies (PRESS) guideline checklist [29].

#### Study records and data management

All study records will be consolidated in a Microsoft® Excel® spreadsheet to ensure systematic organisation and ease of access. This spreadsheet will contain all relevant data items, as outlined in the “Data items” section. Data collected using a customised charting tool developed in Google® Forms will be exported to Excel. The specific elements to be extracted are referenced in Table 3 of Appendix 2. All search results will be exported into Zotero v5.0.81 for reference management. Duplicate records will be identified and removed using Zotero’s built-in duplicate detection tool, followed by manual verification. A Zotero library will be created for the review and organised according to the screening stages.

Two reviewers (NN and VD) will independently perform title and abstract screening using the Google Forms tool. Full-text screening will be conducted for all studies that appear eligible. If abstracts are unavailable, the full article will be assessed directly. Any disagreements during the screening process will be resolved through discussion or by consulting a third reviewer (CB).

Calibration exercises will be conducted before formal screening using a random sample of 10 studies. These exercises aim to ensure consistency in study selection, data extraction, and risk of bias assessment. Inter-rater reliability will be measured using Cohen’s Kappa statistic (κ) [30]. Based on calibration results, screening and extraction tools will be refined to improve consistency and reliability.

**Fig. 1:**
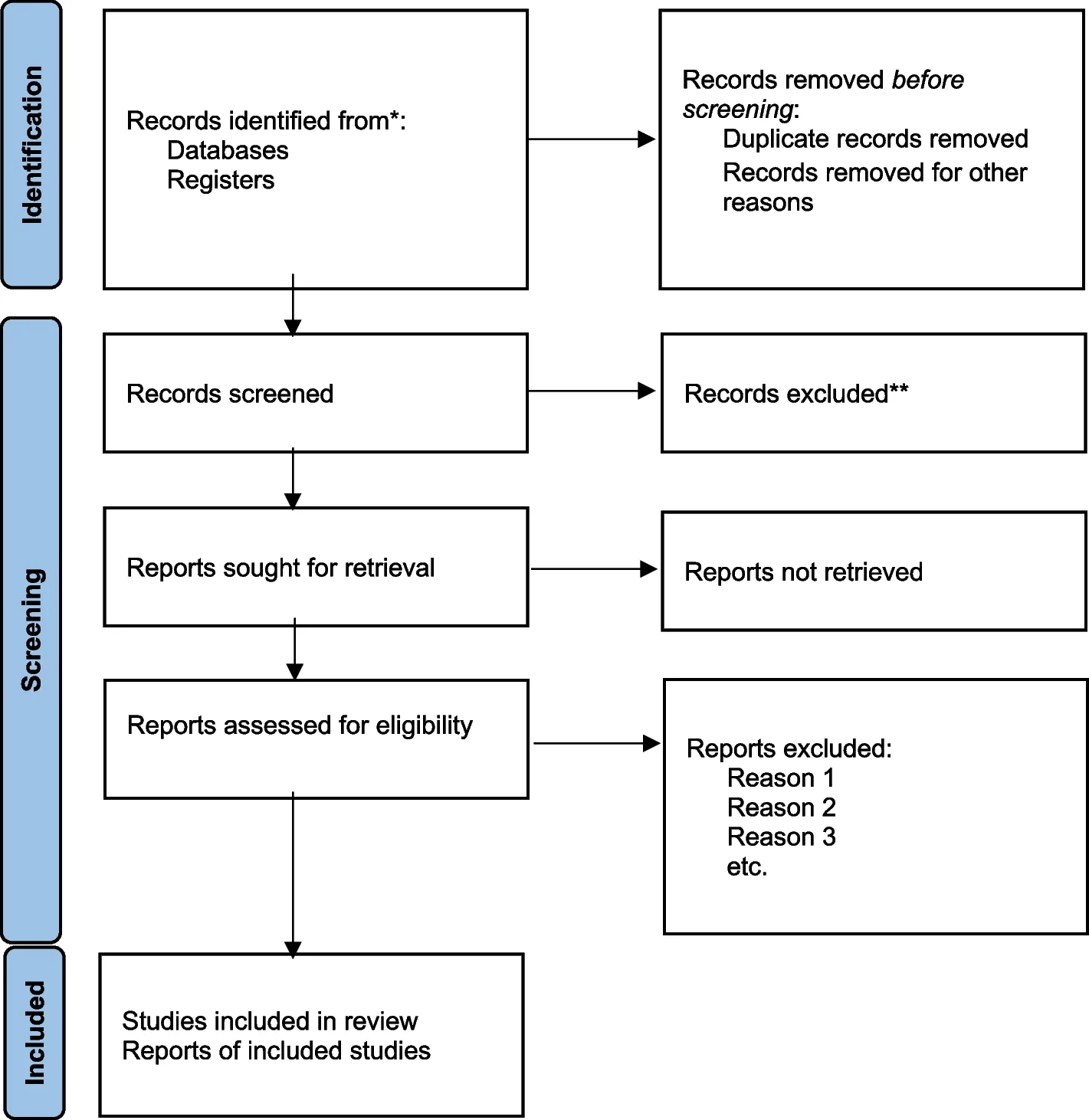
PRISMA flow diagram illustrating the planned study selection process for a systematic review of the impact of LDA on maternal Hb levels [24]

Screening decisions and outcomes will be documented systematically. The study selection process, including reasons for exclusion, will be illustrated using a PRISMA 2020 flow diagram (Fig. 1).

### Data extraction

As illustrated in Table 3 of Appendix 2, a data charting tool (Google Forms) will be employed to extract and process information from each selected study. To ensure comprehensive data collection on relevant aspects of the study, the form will be piloted, and continually updated should the need arise.

Data items to be extracted include the following:

- Study characteristics: authors, year of publication, country, study design, sample size, setting, and duration of follow-up.
- Population details: participant age/demographics, gestational age at enrolment, and relevant baseline characteristics as reported by the study.
- Intervention and comparator: details on low-dose aspirin use, including dose, timing of initiation, duration, and comparator group.
- Outcomes: reported maternal outcomes relevant to this review, including change in Hb levels, incidence or severity of anaemia, development of hypertensive disorders of pregnancy, fetal growth parameters, birth weight, and preterm birth. For each outcome, the timing, method of measurement, and diagnostic criteria as reported in the original study will be recorded.
- Potential confounders: reported information on iron supplementation, nutritional status, chronic condition (e.g. HIV, malaria), inflammation or infection, and concomitant medications where available.
- Other variables: source of study funding, reported conflicts of interest, and any additional notes relevant to risk of bias.

Two reviewers will independently extract data from each included study to ensure accuracy and completeness. Discrepancies will be resolved through discussion, with consultation of a third reviewer where necessary.

#### Risk of bias in individual studies

The risk of bias for individual studies will be assessed using appropriate tools for each study type:

- For randomised controlled trials (RCTs), we will use the Cochrane Risk of Bias 2.0 (RoB 2) tool. This tool assesses bias across five domains: randomisation process, deviations from intended interventions, missing outcome data, measurement of the outcome, and selection of the reported result [31]. Each domain will receive one of the following judgments: low risk of bias, some concerns and high risk of bias.
- For observational studies, we will use the Risk Of Bias In Non-randomised Studies of Interventions-I (ROBINS-I). This tool is designed to assess risk of bias in non-randomised studies by evaluating potential confounding and other biases specific to observational designs, including biases due to selection, classification of interventions, deviations from intended interventions, missing data, outcome measurement, and selective reporting [32]. Each domain is rated as: low risk of bias, moderate risk of bias, critical risk of bias and no information.

Risk of bias will be independently assessed by two reviewers using the appropriate validated tools for each study design. Any disagreements will be resolved through discussion, with involvement of a third reviewer to reach consensus.

The overall risk-of-bias assessments will be presented in:

- Summary tables
- Graphical displays (e.g. traffic-light plots or domain-level bar charts).

### Assessment of publication bias

Publication bias will be assessed where a sufficient number of studies are available. If ≥ 10 studies are included in a quantitative synthesis, publication bias will be examined using funnel plots and Egger’s regression test.

When fewer than 10 studies are included, publication bias and small-study effects will be assessed using the Doi plot and the Luis Furuya-Kanamori (LFK) index, which have demonstrated improved sensitivity in meta-analyses with a limited number of studies [33]. An LFK index between − 1 and + 1 will be interpreted as indicating no major asymmetry.

### Data analysis and synthesis

Where studies are sufficiently similar in terms of study design, population characteristics, interventions, and outcome definitions, a meta-analysis will be conducted. Randomised controlled trials and observational studies will be analysed separately to preserve methodological consistency.

A random-effects model will be used as the primary analytical approach in MetaXL®, as clinical and methodological heterogeneity is anticipated across studies, including differences in study design, participant characteristics, baseline anaemia status, aspirin dose and timing of initiation, outcome definitions, and methods of outcome assessment. This approach assumes that true effect sizes may vary between studies and therefore provides more conservative and generalisable estimates.

A fixed-effect model will only be considered where studies are judged to be clinically and methodologically homogeneous and where statistical heterogeneity is low (*I*^2^ ≤ 50%), based on clinical judgement.

For continuous outcomes (e.g. change in Hb levels, birth weight, or fetal biometric measurements), pooled effects will be expressed as mean differences (MDs) when outcomes are reported on the same scale, or standardised mean differences (SMDs) when different measurement scales are used. For dichotomous outcomes (e.g. incidence of anaemia or HDP), pooled estimates will be presented as risk ratios (RRs) or odds ratios (ORs), with 95% confidence intervals (CIs). Statistical heterogeneity will be assessed using the *I*^2^ statistic and visual inspection of forest plots.

Where substantial heterogeneity is detected (*I*^2^ > 50%), potential sources of heterogeneity will be explored through predefined subgroup analyses, including:

- Study design (randomised-controlled trials vs observational studies)
- Aspirin dose and timing of initiation
- Baseline anaemia status
- Geographic region or healthcare setting

Sensitivity analyses will be conducted to assess the robustness of pooled estimates by excluding studies at high risk of bias or with small sample sizes [34].

If quantitative synthesis is not appropriate due to substantial heterogeneity or insufficient data, findings will be summarised using a structured narrative synthesis, organised by study design, population characteristics, intervention details, and direction and magnitude of effects.

### Confidence in cumulative evidence

The certainty of evidence for each key outcome will be assessed using the Grading of Recommendations Assessment, Development and Evaluation (GRADE) approach. The GRADE framework evaluates evidence across five domains: risk of bias, inconsistency, indirectness, imprecision, and publication bias.

For RCTs, evidence for each outcome will initially be rated as high certainty and downgraded by one or more levels if limitations are identified in any of the GRADE domains. For observational studies, evidence will initially be rated as low certainty and may be upgraded if there is evidence of a large magnitude of effect, a dose-response relationship, or if all plausible residual confounding would reduce a demonstrated effect [35].

Certainty of evidence will be categorised as high, moderate, low, or very low. The assessment for GRADE will be conducted separately for each outcome, and the results will be presented in Summary of Findings (SoF) tables, generated using GRADEpro GDT.

## Discussion

There is robust evidence supporting the use of LDA for the prevention of PE in high-risk pregnancies. For instance, a major systematic review of 28 trials demonstrated that initiating LDA at or before 16 weeks significantly reduces the risk of both preterm and term PE, as well as intrauterine growth restriction [36]. Therefore, clinical guidelines recommend daily LDA (81 mg) from the first or early second trimester for women at elevated risk of PE [37, 38]. These guidelines report no major increase in adverse bleeding events; however, the extent to which bleeding contributes to anaemia remains unclear. In LMICs, where baseline anaemia prevalence is already high, even mild, or subclinical bleeding may increase anaemia risk [39].

Most studies evaluating LDA have primarily focused on PE prevention, with haematologic outcomes reported only as bleeding rather than changes in Hb [40–42]. One exception is the secondary analysis of the ASPIRIN trial by Jessani et al. (2021), which measured Hb at the first trimester and at 26–30 weeks and found no significant difference between LDA and placebo groups [43]. However, few studies have adjusted for key confounding factors such as nutritional status, iron supplementation, and infectious or chronic diseases, particularly in resource-limited settings. Evidence from malaria-endemic regions such as the Democratic Republic of Congo suggests that malaria infection modifies the effect of LDA on pregnancy outcomes, including perinatal mortality, indicating that infectious diseases may influence aspirin’s efficacy during pregnancy [44]. In these contexts, infections may blunt LDA’s vascular benefits or alter its haematologic effects. Although some observational studies reported increased risk of PPH with LDA use, they did not address long-term effects on maternal iron stores or red-cell mass [15, 45]. Mechanistic studies further suggest that aspirin metabolites may exert mild iron-chelating effects, potentially impairing iron absorption and contributing to IDA [16–18].

The relationship between LDA, iron homeostasis, and maternal Hb is further complicated in LMICs, where nutritional deficiencies, low supplementation adherence, and chronic infections are common. Iron deficiency, HIV, tuberculosis, malaria, and other inflammatory conditions contribute to functional iron deficiency through hepcidin-mediated pathways and impaired erythropoiesis [46–48]. Tuberculosis induces a pro-inflammatory state associated with reduced erythropoiesis, while malaria disrupts red blood cell turnover and increases iron demand through haemolysis [49, 50]. In addition, structural and health system barriers, including late antenatal booking, limited diagnostic capacity, inconsistent availability of iron supplements or aspirin, and fragmented continuity of care, can delay LDA initiation, hinder Hb monitoring, and amplify anaemia risk [51, 52]. Overall, these factors may independently affect maternal Hb or modify the response to LDA, making it challenging to isolate aspirin’s effect in studies that do not adequately control for these variables.

Given these evidence gaps, a systematic review focused specifically on the effect of LDA on maternal Hb levels is warranted. The review will synthesise available data, examine population-specific confounders, and clarify whether LDA has any clinically meaningful impact on maternal Hb or anaemia risk. Findings from this work may guide clinical decision-making—such as whether enhanced Hb monitoring is advisable for women receiving LDA—and inform public health strategies in regions with high anaemia burdens. Furthermore, by identifying gaps in current evidence, this review may support future research aimed at optimising both PE prevention strategies and maternal haematologic health.

### Registration

The details about the registration of this protocol, including the registry name (Nokwethemba Ngcobo) and PROSPERO number (CRD42024556220), were registered on 22 June 2024. Minor editorial amendments were made during peer review; however, no methodological changes were introduced

### Amendments

Any necessary amendments to this protocol will be documented independently, including the date, nature of the change, and rationale behind it.

## Supplementary Information


Additional file 1. PRISMA-P checklist.Additional file 2. Fee waiver letter.

## Data Availability

All data collected or analysed during this study will be included in the published systematic review article and will be made available upon request.

## Appendix 1

**Table 2:** Search strategy piloted in PubMed

Database used	Search terms
PubMed	(("Aspirin"[MeSH Terms] OR "low-dose aspirin" OR "acetylsalicylic acid") AND ("Anemia"[MeSH Terms] OR "haemoglobin" OR "hemoglobin" OR "blood iron levels" OR "iron deficiency"[MeSH Terms]) AND ("Pregnancy"[MeSH Terms] OR "pregnant women" OR "maternal" OR "pregnancy complications"[MeSH Terms] OR "hypertensive disorder of pregnancy"[MeSH Terms] OR "preeclampsia"[MeSH Terms] OR "pregnancy outcomes"[MeSH Terms] OR "obstetric hemorrhage"[MeSH Terms] OR "postpartum hemorrhage"[MeSH Terms]))

The final search will be conducted during the review process. Search results will be reported in the completed systematic review

## Appendix 2

**Table 3:** Data charting table

Study identification
Authors
Year of publication
Title of the study
Journal name
Study characteristics
Study design
Sample size
Study location
Duration of follow-up
Participant characteristics
Population
Inclusion and exclusion criteria
Gestational age at the start of aspirin usage
Demographics
Intervention details
Dosage of LDA
Timing of initiation
Duration of aspirin use
Comparison group details
Outcomes measured
Haemoglobin levels (baseline and follow-up)
Incidence of anaemia
Changes in haemoglobin levels over time
Pregnancy outcomes
Incidence of preeclampsia
Fetal growth rate
Birth weight
Other relevant outcomes (e.g. preterm birth, stillbirth)
Results
Main findings related to haemoglobin levels
Main findings related to anaemia
Associations between aspirin usage and pregnancy outcomes
Statistical significance and effect sizes
Risk of bias and quality assessment
Methodological quality of the studies
Confounding factors considered

## Abbreviations

LDA: Low-dose aspirin
Hb: Hemoglobin
PE: Preeclampsia
IUGR: Intrauterine growth restriction
PPH: Postpartum haemorrhage
WHO: World Health Organization
MD: Mean difference
SMD: Standardised mean difference
RR: Risk ratio
OR: Odds ratio
LMICs: Low- and middle-income countries

## References

[1] Vane JR, Botting RM. The mechanism of action of aspirin. Thromb Res. 2003;110(5–6):255–8. 10.1016/S0049-3848(03)00379-7. 14592543

[2] Afolabi BB, Babah OA, Adeyemo TA, Odukoya OO, Ezeaka CV, Nwaiwu O, et al. Low-dose aspirin for preventing intrauterine growth restriction and pre-eclampsia in sickle cell pregnancy (PIPSICKLE): a randomised controlled trial (study protocol). BMJ Open. 2021;11(8):e047949. 10.1136/bmjopen-2020-047949. 34389570 PMC8365818

[3] Jung J, Rahman MM, Rahman MS, Swe KT, Islam MR, Rahman MO, et al. Effects of hemoglobin levels during pregnancy on adverse maternal and infant outcomes: a systematic review and meta-analysis. Ann N Y Acad Sci. 2019;1450(1):69–82. 10.1111/nyas.14112. 31148191

[4] Shah S. Hypertensive disorders in pregnancy. In: Sachdeva M, Miller I, editors. Obstetric and Gynecologic Nephrology: Women’s health issues in the patient with kidney disease. Cham: Springer International Publishing; 2019 Oct; 19:p.11–23. Available from: 10.1007/978-3-030-25324-0.

[5] Jena MK, Sharma NR, Petitt M, Maulik D, Nayak NR. Pathogenesis of preeclampsia and therapeutic approaches targeting the placenta. Biomolecules. 2020;10(6):953. 10.3390/biom10060953. 32599856 PMC7357118

[6] Kahramanoglu Ö, Schiattarella A, Demirci O, Sisti G, Ammaturo FP, Trotta C, et al. Preeclampsia: state of art and future perspectives. A special focus on possible preventions. J Obstet Gynaecol. 2022;42(5):766–77. 10.1080/01443615.2022.2048810. 35469530

[7] Atallah A, Lecarpentier E, Goffinet F, Doret-Dion M, Gaucherand P, Tsatsaris V. Aspirin for prevention of preeclampsia. Drugs. 2017;77(17):1819–31. 10.1007/s40265-017-0823-0. 29039130 PMC5681618

[8] Giménez-Bastida JA, Boeglin WE, Boutaud O, Malkowski MG, Schneider C. Residual cyclooxygenase activity of aspirin-acetylated COX-2 forms 15R-prostaglandins that inhibit platelet aggregation. FASEB J. 2019;33(1):1033–41. 10.1096/fj.201801018R. 30096040 PMC6355089

[9] Lee A, Chow L, Skeith L, Nicholas JA, Poon MC, Poole AW, et al. Platelet membrane procoagulation in preeclampsia. Blood. 2020 Nov 5;136(Suppl 1):7. Available from: https://ashpublications.org/blood/article/136/Supplement%201/7/472360/Platelet-Membrane-Procoagulation-in-Preeclampsia.

[10] Sahin S, Ozakpinar O, Eroglu M, Tetik S. Platelets in preeclampsia: function and role in the inflammation. Clin Exp Health Sci. 2014 Apr 1;4(1):111–16. Available from: http://www.clinexphealthsci.com/sayilar/108/buyuk/111-1161.pdf.

[11] Ren Y, Zhao Y, Yang X, Shen C, Luo H. Application of low dose aspirin in pre-eclampsia. Front Med (Lausanne). 2023 Mar 8;10:1111371. Available from: https://www.frontiersin.org/articles/10.3389/fmed.2023.1111371. 10.3389/fmed.2023.1111371 PMC10030847 36968826

[12] Ohuma EO, Young MF, Martorell R, Ismail LC, Pena-Rosas JP, Purwar M, et al. International values for haemoglobin distributions in healthy pregnant women. eClinicalMedicine. 2020 Dec 1;29:100651. Available from: 10.1016/j.eclinm.2020.100651. PMC7788439 33437954

[13] Prakash S, Yadav K. Maternal anemia in pregnancy: an overview. Int J Pharma Pharma Res Hum. 2015 Oct;4(3):164–79. Available from: https://ijppr.humanjournals.com/2015/10/.

[14] World Health Organization. Anaemia [Internet]. Geneva: WHO; 2023 [cited 2025 Jun 20]. Available from: https://www.who.int/news-room/fact-sheets/detail/anaemia.

[15] Hastie R, Tong S, Wikström AK, Sandström A, Hesselman S, Bergman L. Aspirin use during pregnancy and the risk of bleeding complications: a Swedish population-based cohort study. Am J Obstet Gynecol. 2021;224(1):95.e1-95.e12. 10.1016/j.ajog.2020.09.042. 32687818

[16] Kontoghiorghes GJ. The puzzle of aspirin and iron deficiency: the vital missing link of the iron-chelating metabolites. Int J Mol Sci. 2024;25(10):5150. 10.3390/ijms25105150. 38791185 PMC11121054

[17] Kontoghiorghes GJ, Kolnagou A. Molecular factors and mechanisms affecting iron and other metal excretion or absorption in health and disease. The role of natural and synthetic chelators. Curr Med Chem. 2005;12(23):2695–709. Available from: https://www.ingentaconnect.com/content/ben/cmc/2005/00000012/00000023/art00004. 10.2174/092986705774463030 16305466

[18] Pitt CG, Gupta G. The design and synthesis of chelating agents for the treatment of iron overload in Cooley’s anemia. In: Development of iron chelators for clinical use*.* Washington (DC): Department of Health, Education, and Welfare (DHEW); 1976. p. 137–68. Available from: https://books.google.com/books?hl=en&lr=&id=i_RsAAAAMAAJ&oi=fnd&pg=PA137.

[19] Bienstock JL, Eke AC, Hueppchen NA. Postpartum hemorrhage. N Engl J Med. 2021 Apr 29;384(17):1635–45. Available from: http://www.nejm.org/doi/10.1056/NEJMra1513247. 10.1056/NEJMra1513247 PMC10181876 33913640

[20] Say L, Chou D, Gemmill A, Tunçalp Ö, Moller AB, Daniels J, et al. Global causes of maternal death: a WHO systematic analysis. Lancet Glob Health. 2014 Jun;2(6):e323–33. Available from: https://www.sciencedirect.com/science/article/pii/S2214109X1470227X. 10.1016/S2214-109X(14)70227-X 25103301

[21] Ahrens KA, Silver RM, Mumford SL, Sjaarda LA, Perkins NJ, Wactawski-Wende J, et al. Complications and safety of preconception low-dose aspirin among women with prior pregnancy losses. Obstet Gynecol. 2016 Apr 1;127(4):689–98. Available from: 10.1097/AOG.0000000000001331. PMC4805457 26959198

[22] Kupka E, Roberts JM, Mahdy ZA, Escudero C, Bergman L, De Oliveira L, et al. Aspirin for preeclampsia prevention in low- and middle-income countries: mind the gaps. AJOG Glob Rep. 2024;100352. Available from: https://www.sciencedirect.com/science/article/pii/S2666577824000467. 10.1016/j.xagr.2024.100352 PMC11061325 38694484

[23] Nugroho FL, Ariningtyas ND, Rezkita YAA, Budinurdjaja P, Anas M. Relationship of anemia in pregnancy with postpartum hemorrhage in Jombang Regional Hospital. Indones J Med Sci Public Health. 2020;1(1):1–6. Available from: https://ijmpjournal.org/index.php/ijmp/article/view/10.

[24] Page MJ, McKenzie JE, Bossuyt PM, Boutron I, Hoffmann TC, Mulrow CD, et al. The PRISMA 2020 statement: an updated guideline for reporting systematic reviews. BMJ. 2021;372:n71. 10.1136/bmj.n71. 33782057 PMC8005924

[25] World Health Organization. Guideline on haemoglobin cutoffs to define anaemia in individuals and populations [Internet]. Geneva: World Health Organization; 2024 Mar 5. Available from: https://books.google.com/books?hl=en&lr=&id=uaYOEQAAQBAJ&oi=fnd&pg=PR3. 38530913

[26] Brown MA, Magee LA, Kenny LC, Karumanchi SA, McCarthy FP, Saito S, et al. Hypertensive disorders of pregnancy: ISSHP classification, diagnosis, and management recommendations for international practice. Hypertension. 2018;72(1):24–43. 10.1161/HYPERTENSIONAHA.117.10803. 29899139

[27] DeMars MM, Perruso C. MeSH and text-word search strategies: precision, recall, and their implications for library instruction. J Med Libr Assoc. 2022 Jan 1;110(1). Available from: https://jmla.pitt.edu/ojs/jmla/article/view/1283. 10.5195/jmla.2022.1283 PMC8830400 35210959

[28] Mahood Q, Van Eerd D, Irvin E. Searching for grey literature for systematic reviews: challenges and benefits. Res Synth Methods. 2014;5(3):221–34. 10.1002/jrsm.1106. 26052848

[29] McGowan J, Sampson M, Salzwedel DM, Cogo E, Foerster V, Lefebvre C. PRESS peer review of electronic search strategies: 2015 guideline statement. J Clin Epidemiol. 2016 Jul 1;75:40–6. Available from: https://linkinghub.elsevier.com/retrieve/pii/S0895435616000585. 10.1016/j.jclinepi.2016.01.021 27005575

[30] Bramer WM, De Jonge GB, Rethlefsen ML, Mast F, Kleijnen J. A systematic approach to searching: an efficient and complete method to develop literature searches. J Med Libr Assoc. 2018 Oct 1;106(4). Available from: http://jmla.pitt.edu/ojs/jmla/article/view/283. 10.5195/jmla.2018.283 PMC6148622 30271302

[31] Higgins JPT, Altman DG, Gotzsche PC, Juni P, Moher D, Oxman AD, et al. The Cochrane Collaboration’s tool for assessing risk of bias in randomised trials. BMJ. 2011;343:d5928. 10.1136/bmj.d5928. 22008217 PMC3196245

[32] Sterne JA, Hernán MA, Reeves BC, Savović J, Berkman ND, Viswanathan M, et al. ROBINS-I: a tool for assessing risk of bias in non-randomised studies of interventions. BMJ. 2016 Oct 1;355:i4919. Available from: https://www.bmj.com/lookup/doi/10.1136/bmj.i4919. 10.1136/bmj.i4919 PMC5062054 27733354

[33] Furuya-Kanamori L, Barendregt JJ, SAR. A new improved graphical and quantitative method for detecting bias in meta-analysis. Int J Evid Based Healthc. 2018 Dec;16(4):195–203. Available from: https://journals.lww.com/01787381-201812000-00003. 10.1097/XEB.0000000000000141 29621038

[34] Bianchim MS, McNarry MA, Larun L, Mackintosh KA. Calibration and validation of accelerometry to measure physical activity in adult clinical groups: a systematic review. Prev Med Rep. 2019 Dec 1;16:101001. Available from: https://linkinghub.elsevier.com/retrieve/pii/S221133551930172X. 10.1016/j.pmedr.2019.101001 PMC6931234 31890467

[35] Schünemann H, Brożek J, Guyatt G, Oxman A. The GRADE handbook [Internet]. Cochrane Collaboration; 2013 Oct 4 [cited 2025 Jun 20]. Available from: https://hero.epa.gov/hero/index.cfm/reference/details/reference_id/10284249.

[36] Wang W, Chang G. Evaluation of low-dose aspirin on pregnancy outcomes: a systematic review and meta-analysis. Arch Iran Med. 2025;28(4):225. 40382694 10.34172/aim.33275 PMC12085792

[37] Committee on Obstetric Practice. ACOG committee opinion no. 743: low-dose aspirin use during pregnancy. Obstet Gynecol. 2018;132(1):e44-52. 10.1097/AOG.0000000000002580. 29939940

[38] US Preventive Services Task Force, Davidson KW, Barry MJ, Mangione CM, Cabana M, Caughey AB, et al. Aspirin use to prevent preeclampsia and related morbidity and mortality: US Preventive Services Task Force recommendation statement. JAMA. 2021 Sep 28;326(12):1186–91. Available from: https://jamanetwork.com/journals/jama/fullarticle/2784499. 10.1001/jama.2021.14781 34581729

[39] Balarajan Y, Ramakrishnan U, Özaltin E, Shankar AH, Subramanian SV. Anaemia in low-income and middle-income countries. Lancet. 2011;378(9809):2123–35. 21813172 10.1016/S0140-6736(10)62304-5

[40] Rolnik DL, Wright D, Poon LC, O’Gorman N, Syngelaki A, de Paco Matallana C, et al. Aspirin versus placebo in pregnancies at high risk for preterm preeclampsia. N Engl J Med. 2017;377(7):613–22. Available from: https://www.nejm.org/doi/abs/10.1056/NEJMoa1704559. 10.1056/NEJMoa1704559 28657417

[41] Duley L, Meher S, Hunter KE, Seidler AL, Askie LM. Antiplatelet agents for preventing pre-eclampsia and its complications. Cochrane database Syst Rev. 2019;(10):CD004659. Available from: https://www.cochranelibrary.com/cdsr/doi/10.1002/14651858.CD004659.pub3/abstract. 10.1002/14651858.CD004659.pub3 PMC6820858 31684684

[42] Roberge S, Bujold E, Nicolaides KH. Aspirin for the prevention of preterm and term preeclampsia: systematic review and meta-analysis. Am J Obstet Gynecol. 2018 Mar;218(3):287–293.e1. Available from: https://linkinghub.elsevier.com/retrieve/pii/S0002937817323268. 10.1016/j.ajog.2017.11.561 29138036

[43] Jessani S, Saleem S, Hoffman M, Goudar S, Derman R, Moore J, et al. Association of haemoglobin levels in the first trimester and at 26–30 weeks with fetal and neonatal outcomes: a secondary analysis of the Global Network for Women’s and Children’s Health’s ASPIRIN Trial. BJOG. 2021 Aug;128(9):1487–96. Available from: https://obgyn.onlinelibrary.wiley.com/doi/10.1111/1471-0528.16676. 10.1111/1471-0528.16676 PMC8286300 33629490

[44] Bauserman M, Leuba SI, Hemingway-Foday J, Nolen TL, Moore J, McClure EM, et al. The efficacy of low-dose aspirin in pregnancy among women in malaria-endemic countries. BMC Pregnancy Childbirth. 2022 Dec;22(1):303. Available from: https://bmcpregnancychildbirth.biomedcentral.com/articles/10.1186/s12884-022-04652-9. 10.1186/s12884-022-04652-9 PMC8994890 35399060

[45] Souter V, Painter I, Sitcov K, Khalil A. Propensity score analysis of low‐dose aspirin and bleeding complications in pregnancy. Ultrasound Obstet Gynecol. 2024;63(1):81–7. 37674400 10.1002/uog.27472

[46] Waldvogel-Abramowski S, Waeber G, Gassner C, Buser A, Frey BM, Favrat B, et al. Physiology of iron metabolism. Transfus Med Hemother. 2014 Jun;41(3):213–21. Available from: https://karger.com/TMH/article/doi/10.1159/000362888. 10.1159/000362888 PMC4086762 25053935

[47] Obeagu EI, Obeagu GU, Ukibe NR, Oyebadejo SA. Anemia, iron, and HIV: decoding the interconnected pathways: a review. Medicine. 2024 Jan 12;103(2):e36937. Available from: https://journals.lww.com/md-journal/fulltext/2024/01120/anemia,_iron,_and_hiv__decoding_the_interconnected.51.aspx. 10.1097/MD.0000000000036937 PMC10783375 38215133

[48] Cao G, Wang Y, Wu Y, Jing W, Liu J, Liu M. Prevalence of anemia among people living with HIV: a systematic review and meta-analysis. eClinicalMedicine. 2022 Feb;44:101283. Available from: https://linkinghub.elsevier.com/retrieve/pii/S258953702200013X. 10.1016/j.eclinm.2022.101283 PMC8803600 35128369

[49] Mekenye OJ. Profile of haematological indices among pulmonary tuberculosis patients attending Kisii teaching and referral hospital, Kisii Kenya [PhD Thesis]. Kisii University; 2024 [cited 2025 May 22]. Available from: http://repository.kisiiuniversity.ac.ke:8080/xmlui/handle/123456789/6787.

[50] Santen S van. Iron homeostasis in inflammation, malaria and pregnancy [Internet] [PhD Thesis]. Nijmegen: Radboud University; 2015 [cited 2025 May 22]. Available from: https://repository.ubn.ru.nl/bitstream/handle/2066/141369/MMUBN000001_107296662X.pdf.

[51] King SE. Relations between antenatal quality of care and iron folic acid adherence: a multi-country study [Internet] [PhD Thesis]. Baltimore: Johns Hopkins University; 2022 [cited 2025 May 22]. Available from: https://jscholarship.library.jhu.edu/bitstream/handle/1774.2/67486/KING-DISSERTATION-2022.pdf?sequence=1.

[52] Galloway R, McGuire J. Determinants of compliance with iron supplementation: supplies, side effects, or psychology? Soc Sc Med. 1994 Aug 1;39(3):381–90. Available from: https://www.sciencedirect.com/science/article/pii/027795369490135X. 10.1016/0277-9536(94)90135-x 7939855

