# Supplementary material for: Impact of low-dose aspirin on maternal haemoglobin levels during pregnancy: a protocol for a systematic review and meta-analysis
Source: Syst Rev. 2026 Jun 19;15:235. doi: 10.1186/s13643-026-03219-5 (PMC13523184; doi:10.1186/s13643-026-03219-5)
Supplement: Supplementary file 2 [file 13643_2026_3219_MOESM2_ESM.docx]

Dear Madam/Sir

# Waiver of publication fees

Thank you for your consideration of a waiver of publication costs for the article submitted.

This letter serves to confirm that Ms Nokwethemba Ngcobo (Student number 220024353) is a full-time registered postgraduate for her Masters of Medical Sciences degree in the College of Health Sciences, University of KwaZulu-Natal, Durban, South Africa. She has been awarded the College of Health Sciences bursary that covers her fees and the balance is for her subsistence only.

As far as I am aware, she is not in receipt of any additional funding for herself or the research project. I believe her research endeavour is important, particularly in the context of the high prevalence of maternal mortality in South Africa and hope that you will consider her application favourably.

Sincerely,

# Professor Anil Chuturgoon

**Dean of Research: College of Health Sciences**

**Postal Address:** 1st Floor George Campbell Building, Howard College Campus, UKZN, Durban 4000, South Africa

**Telephone:** +27 (0) 31 260 4404 **Facsimile:** +27 (0) 31 260 1824 **Email:** [CHUTUR@ukzn.ac.za](mailto:CHUTUR@ukzn.ac.za) **Website:** [www.ukzn.ac.za](http://www.ukzn.ac.za/)
